# Intestinal flora imbalance affects bile acid metabolism and is associated with gallstone formation

**DOI:** 10.1186/s12876-020-01195-1

**Published:** 2020-03-06

**Authors:** Qiang Wang, Chenjun Hao, Wenchao Yao, Defu Zhu, Haifeng Lu, Long Li, Biao Ma, Bei Sun, Dongbo Xue, Weihui Zhang

**Affiliations:** Department of General Surgery, Laboratory of Hepatosplenic Surgery, Ministry of Education, The First Affiliated Hospital of Harbin Medical University, Youzheng Street 23, Harbin, 150001 China

**Keywords:** Gut microbiota, Gallstone, Bile acid, BSH, 16S rRNA gene sequencing

## Abstract

**Background:**

The gut microbiota participates in the metabolism of substances and energy, promotes the development and maturation of the immune system, forms the mucosal barrier, and protects the host from pathogen attacks. Although the pathogenesis of cholesterol gallstones is still not clear, studies have suggested that gut microbiota dysbiosis plays an important role in their formation.

**Methods:**

Microbial DNA from faeces of normal control patients and those of patients with calculi was subjected to 16S rRNA gene sequencing to detect gene expression changes in intestinal microbes. ELISA kits were used to measure free bile acids, secondary bile acids and coprostanol according to the manufacturer’s instructions. The relationship between flora and their metabolites was then analysed.

**Results:**

In the gallstone group, the diversity of intestinal bacteria and the abundances of certain phylogroups were significantly decreased (*p* < 0.05), especially *Firmicutes* (*p* < 0.05), the largest phylum represented by the gut microbiota. This study found an increase in free bile acids (*p* < 0.001) and secondary bile acids (*p* < 0.01) in the enterohepatic circulation. Bile salt hydrolase activity was not related to the abundances of BSH-active bacteria. 7a-dehydroxylating gut bacteria were significantly increased (*p* < 0.01), whereas cholesterol-lowering bacteria were significantly reduced (*p* < 0.05). The *Ruminococcus gnavus* group could be used as a biomarker to distinguish the gallstone group from the control group.

**Conclusion:**

We conclude that intestinal flora imbalance affects bile acid and cholesterol metabolism and is associated with gallstone formation.

## Background

Gallstones are a common and frequently occurring disease. Approximately 10–15% of adults have gallstones, which cause great suffering [1]. Most (90%) gallstones are cholesterol gallstones [2]. The pathogenesis of cholesterol gallstones is still not clear, though studies suggest that gut microbiota dysbiosis plays an important role in their formation [3]. The gut microbiota participates in the metabolism of substances and energy, promotes the development and maturation of the immune system, forms the mucosal barrier, and protects the host from pathogen attacks [4]. Generally speaking, there is a symbiotic relationship between the intestinal bacteria and the host, and they maintain the balance of the intestinal microecology [5].. If this balance is disrupted, it may lead to the development of chronic conditions, including colonic inflammation, colon cancer, gallstones [6], and such metabolic diseases as obesity and diabetes [7].

Current studies have shown that changes in gut microbiota are associated with the formation of cholesterol gallstones. Wang et al. [3] investigated the faeces of mice with cholesterol gallstones using 16S ribosomal RNA (16 s rRNA) gene sequencing and found that in the intestinal tract of mice, the *Firmicutes* content and the *Firmicutes*/*Bacteroidetes* (F/B) ratio decreased, the abundances and diversity of the microbiota also significantly decreased, and a lithogenic diet (LD) reshaped the abundances of gut microbiota at different levels. Keren et al. found that in patients with gallstones, the microbial diversity was reduced and the total bile acid concentration in faeces was increased [8]. Bile acid is not only a simple fat emulsion but also an important signalling regulator molecule, as it can activate nuclear farnesoid X receptor (FXR), pregnane X receptor (PXR), vitamin D receptor (VDR), and G-protein-coupled bile acid receptor 1 (GPBAR1, TGR5); it can affect host metabolism of energy, lipids, and glucose; and it can regulate its own synthesis and cholesterol degradation [9]. The gut microbiota can convert cholesterol into coprostanol, which is not easily absorbed or esterified; therefore, it is excreted from the body [10]. The gut microbiota can also shape the development of the intestinal immune system [11, 12].

This study analysed the changes in the structure and function of the gut microbiota and the relationships between the gut microbiota and bile acids and cholesterol in patients with gallstones to provide evidence for the correlation between gut microbiota dysbiosis and gallstone formation.

## Methods

### Study subjects and specimens

There were 30 patients in the gallstone group, including 10 males and 20 females, with an average age of 42.6 ± 12.5 years and body mass index (BMI = body weight/height^2^) of 25.6 ± 4.6 kg/m^2^. The control group included 30 healthy individuals, including 13 males and 17 females, with an average age of 40.5 ± 11.5 years and BMI of 24.7 ± 3.0 kg/m^2^. The average age of the gallstone group was higher than that of the control group; however, the difference was not statistically significant (42.6 ± 12.5 years> 40.5 + 11.5 years, *p* = 0.495 > 0.05). The mean BMI of the gallstone group was higher than that of the control group; however, the difference was not statistically significant (25.6 ± 4.6 kg/m^2^ > 24.7 ± 3.0 kg/m^2^, *p* = 0.367 > 0.05). The subjects in these 2 groups had not taken antibiotics for 3 months, did not have metabolic diseases such as diabetes or hyperthyroidism, did not have cardiovascular diseases such as hypertension or coronary heart disease, did not have gastroenteritis diseases such as gastroenteritis, and did not have a history of gastrointestinal-related surgery before admission. The patients in the gallstone group were confirmed to be free of bile duct stones and gallstone polyps by both colour Doppler ultrasonography and magnetic resonance cholangiopancreatography (MRCP), while the patients in control group were confirmed to have no bile duct stones or gallstone polyps by colour Doppler ultrasonography. The faeces of the subjects at 7:00 in the morning were collected. 1 g of faeces was put into a sterile tube using a sterile spoon. In the same way, we put the feces of each subject into 4 sterile tubes. The tube was sealed with a sealing membrane and rapidly stored in a − 80 °C freezer with a liquid nitrogen tank for later analysis. These analyses include sequencing, free bile acid detection, secondary bile acid detection, and coprostanol detection.

### Illumina MiSeq sequencing

After the extraction of total DNA of the samples, primers were designed according to the conserved region, and sequencing adaptors were attached to the ends of the primers for polymerase chain reaction (PCR) amplification. Purification, quantification, and homogenization of the PCR products were performed to form the sequencing library. The constructed library was first subjected to library quality control. An Illumina HiSeq 2500 was used to sequence the qualified library. The raw image data files were obtained after the high-throughput sequencing (Illumina HiSeq sequencing platform) and were converted to the raw sequenced reads by base-calling analysis. The results were saved in FASTQ (.fq) format, which contained sequence information of the reads and its corresponding quality information.

### Data processing and analysis

#### Data processing

The raw data were first spliced (FLASH [13], version 1.2.11), and quality trimming was applied to the spliced sequence (Trimmomatic [14], version 0.33) to remove the chimaeras (UCHIME [15], version 8.1), in order to get high-quality sequence tags. For paired-end read splicing, FLASH v1.2.11 software was used. According to the minimum overlap length of 10 bp and the allowed maximum mismatch ratio of 0.2 (Default) in the overlap, the reads of each sample were spliced to obtain the spliced sequence, i.e., the raw tags. The operating principle of Trimmomatic is as follows. Trimmomatic is a flexible trimmer for Illumina sequence data. It can be used for quality trimming of double-ended sequencing or single-ended sequencing data in the FASTQ format with the base quality of phred 33 or phred 64 (depending on the Illumina sequencing machine). The parameters were set as follows. The length of the window was 50 bp. If the mean quality value within the window was lower than 20, the back bases were truncated from the window, and the tags whose quality control was shorter than 75% of the length of the tags were filtered to obtain high-quality tag data, i.e., clean tags. The principle of UCHIME is shown in the figure below. In the first step, the query sequence is split into chunks that do not overlap, and then the chunks are compared with the database. The second step is to select the best match for each chunk in the database and ultimately select the 2 best parent sequences. In the third step, the query sequence to be detected is compared with those 2 parent sequences. If there is a sequence in each of the 2 parent sequences whose similarity with the query sequence is over 80%, the query is determined to be a chimaera.

#### Data analysis

Operational taxonomic unit (OTU) analysis clustered sequences at the 97% similarity level (USEARCH [16], version 10.0), and 0.005% of all sequences was used as the threshold to filter OTUs [17]. Species annotation and taxonomic analysis: the 16S:Silva rRNA gene database was selected [18] (Release128, http://www.arb-silva.de). Alpha index analysis: Shannon diversity curves and rank species abundance (RSA) curves [19] (rank abundance curves) were used. The software for these tasks was Mothur [20] version v.1.30 (http://www.mothur.org/). Beta diversity: species diversity matrix was calculated by a variety of algorithms, such as binary Jaccard, Bray–Curtis, and unweighted UniFrac (only for bacteria). The principal coordinate analysis (PCoA) results were plotted using R software. Line discriminant analysis (LDA) effect size (LEfSe) analysis (http://huttenhower.sph.harvard.edu/lefse/), the analysis of significant differences between groups (which can be called biomarker analysis), used LDA to estimate the impact of the abundance of each component (species) on the differences, and a logarithm of LDA score of 4.0 was set as the cut-off for significant differences. The gut microbiota with encoded bile salt hydrolase (BSH) genes were identified by a literature review [21]. The gut microbiota with BSH activity in the gallstone group was selected by referring to the literature and the database of the Human Microbiome Project (https://www.hmpdacc.org).

### Detection of free bile acids, secondary bile acids and coprostanol

According to the manufacturer’s instructions (sino best bio co.ltd), we used ELISA kits to measure the concentration of free bile acids, secondary bile acids and cholesterol in stool separately. Take approximately 100 mg of feces from a sterile tube, wash the feces three times with PBS (final fecal mass: PBS volume = 1: 9), or mash them and centrifuge at 5000×g for 10 min, and take the supernatant for detection. Procedure: 1. The plates were taken out of the aluminium foil bag after 20 min at room temperature. 2. Standard wells and sample wells were set, and different concentrations of the standards were added to standard wells (50 μL/well). 3. Ten microlitres of sample solution was added to the sample well, followed by 40 μL of sample diluent. Blank wells contained no solution. 4. In addition to the blank wells, horseradish peroxidase-labelled detection antibody (100 μL) was added to each standard and sample well. The sample wells were sealed with a plate-sealing membrane and incubated at 37 °C in a water bath or incubator for 60 min. 5. The liquid was removed, the wells were dried on blotting paper, each well was filled with washing solution for 1 min, the washing solution was removed, and the wells were dried on blotting paper. This washing cycle was repeated 5 times. 6. Substrate (50 μL) was added to each well, and the wells were incubated at 37 °C in the dark for 15 min. 7. Stop solution (50 μL) was added to each well. Within 15 min, the optical density (OD) value of each well was measured at 450 nm. The results were used to determine the standard curve. In an Excel worksheet, the standard concentration was used as the abscissa, and the corresponding OD value was used as the ordinate to plot the linear regression curve of the standard. The concentration values for each sample were calculated according to the curve equation.

### Statistics

Data are expressed as the mean ± standard deviation (SD). If the data were normally distributed, mention the groups were compared using the t test. If the data were not normally distributed, the difference was compared using the Mann-Whitney U test. *P* < 0.05 was considered statistically significant. The relationship between tree bile acids, secondary bile acids, and coprostanol with bacteria was separately analysed by Spearman’s correlation.

## Results

### Sequencing data quality assessment

The number of sample sequences in each stage was processed through statistical data to evaluate the data quality. The data is mainly evaluated by counting the sequence number, sequence length, GC content, Q20,Q30 quality values and Effective(%)at each stage (Supplementary material 1).

### Results from the OTU-based analysis

**Fig. 1:**
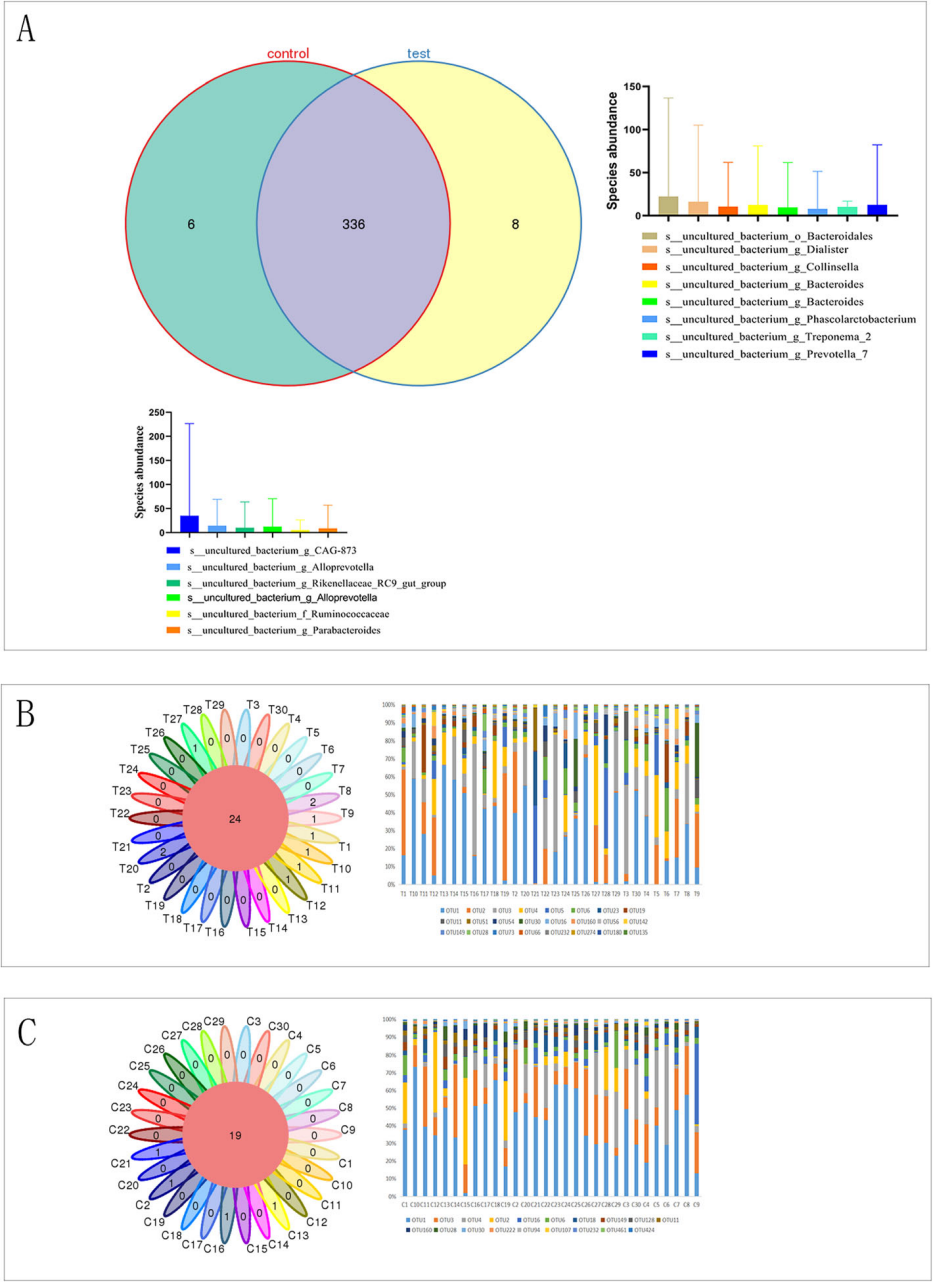
Results of the OTU-based analysis. **a** The gallstone group had 8 unique bacteria, and the control group had 6 unique bacteria. **b** In all, 24 OTUs in the gallstone group were in a public database. **c** In all, 19 OTUs in the control group were in a public database

In all, 336 public OTUs from two groups of intersections are publicly available. The gallstone group had 8 unique bacteria, while the control group had 6 unique bacteria (Fig. 1a). The bacterial species of each sample in the gallstone group were essentially the same, and 24 OTUs were publicly available (Fig. 1b). The OTU of each sample in the control group was also basically the same, and 19 OTUs were in the public database (Fig. 1c). The results showed that the individual differences within the group were small.

### Decreased abundance and diversity of intestinal bacteria in the gallstone group

**Fig. 2:**
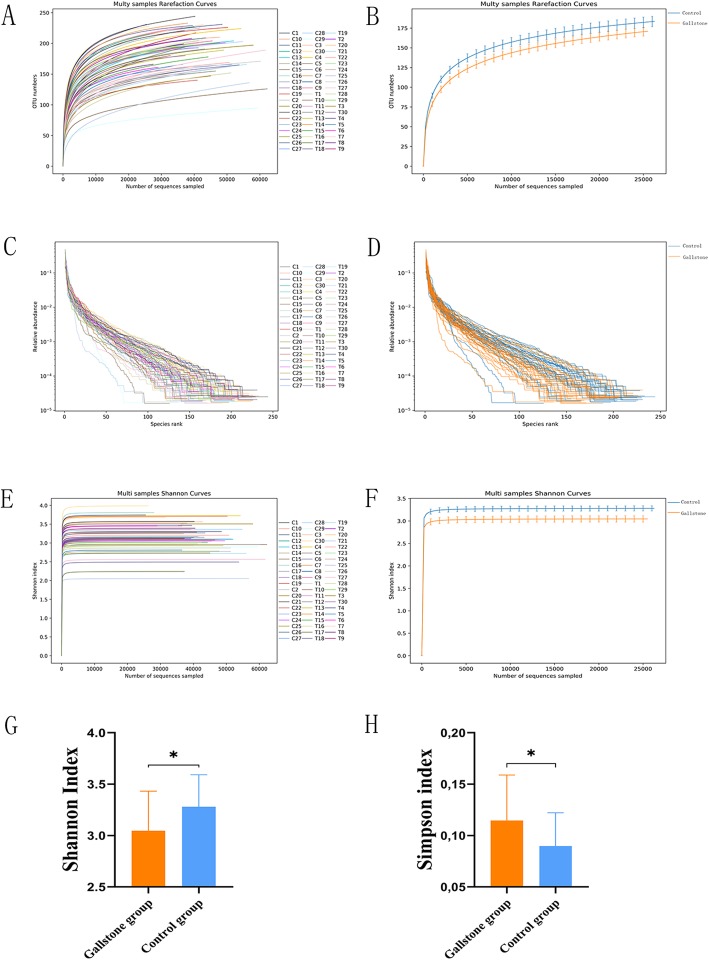
**a** The Rarefaction Curve tends to be flat, which indicates that the sequencing of each sample was sufficient to reflect the species diversity in the sample. **b** The species richness of the gallstone group was less than that of the control group using the same number of sequences. **c** and **d** The rank abundance curve of most samples in the gallstone group was shorter than that in the control group in the horizontal direction, and the shape of the curve was steeper. **e** The curve tends to be flat, which indicates that the amount of sequencing data is large enough to reflect the biological diversity of the sample. **f** and **g** The Shannon index of the gallstone group was significantly smaller than that of the control group (3.04 ± 0.38 < 3.28 ± 0.31, *p* < 0.05). **h** The Simpson index was significantly greater in the gallstone than in the control group (0.11 ± 0.04 > 0.08 ± 0.03, *p* < 0.05). Data are expressed as the mean ± SD, *p < 0.05, **p < 0.01. Statistically significant differences (*p* < 0.05) between the two groups were determined by Student’s t test

The rarefaction curve was used to verify that the amount of sequencing data was sufficient to reflect the species diversity in the sample and that the data could indirectly reflect the abundances of the species in the sample. The results showed that as the number of sequences increased, the curve tended to be flat, indicating that the sequencing of each sample was sufficient to reflect the species diversity in the sample (Fig. 2a) and that the species richness in the gallstone group was lower than that in the control group using the same number of sequences (Fig. 2b). The rank abundance curve was mainly used to explain the abundances and uniformity of the species contained in the sample. The results showed that the rank abundance curve of most samples in the gallstone group was shorter than that in the control group in the horizontal direction, and the shape of the curve was steeper (Fig. 2c), indicating that the species richness in the gallstone group was reduced and that the species uniformity was low (Fig. 2d). The Shannon index was used to reflect the microbial diversity of each sample using different numbers of sequences. As the number of sequences increased, the curve tended to be flat, indicating that the amount of sequencing data was large enough to reflect the biological diversity of the sample (Fig. 2e). Interestingly, the Shannon index of the gallstone group was significantly smaller than that of the control group (3.04 ± 0.38 < 3.28 ± 0.31, *p* = 0.012) (Fig. 2f,g), while the Simpson index was significantly greater in the gallstone group than in the control group (0.11 ± 0.04 > 0.08 ± 0.03, *p* = 0.015), demonstrating a significant reduction in species diversity in the gallstone group (Fig. 2h).

### β-Diversity analysis of the microbial composition in the 2 groups

**Fig. 3:**
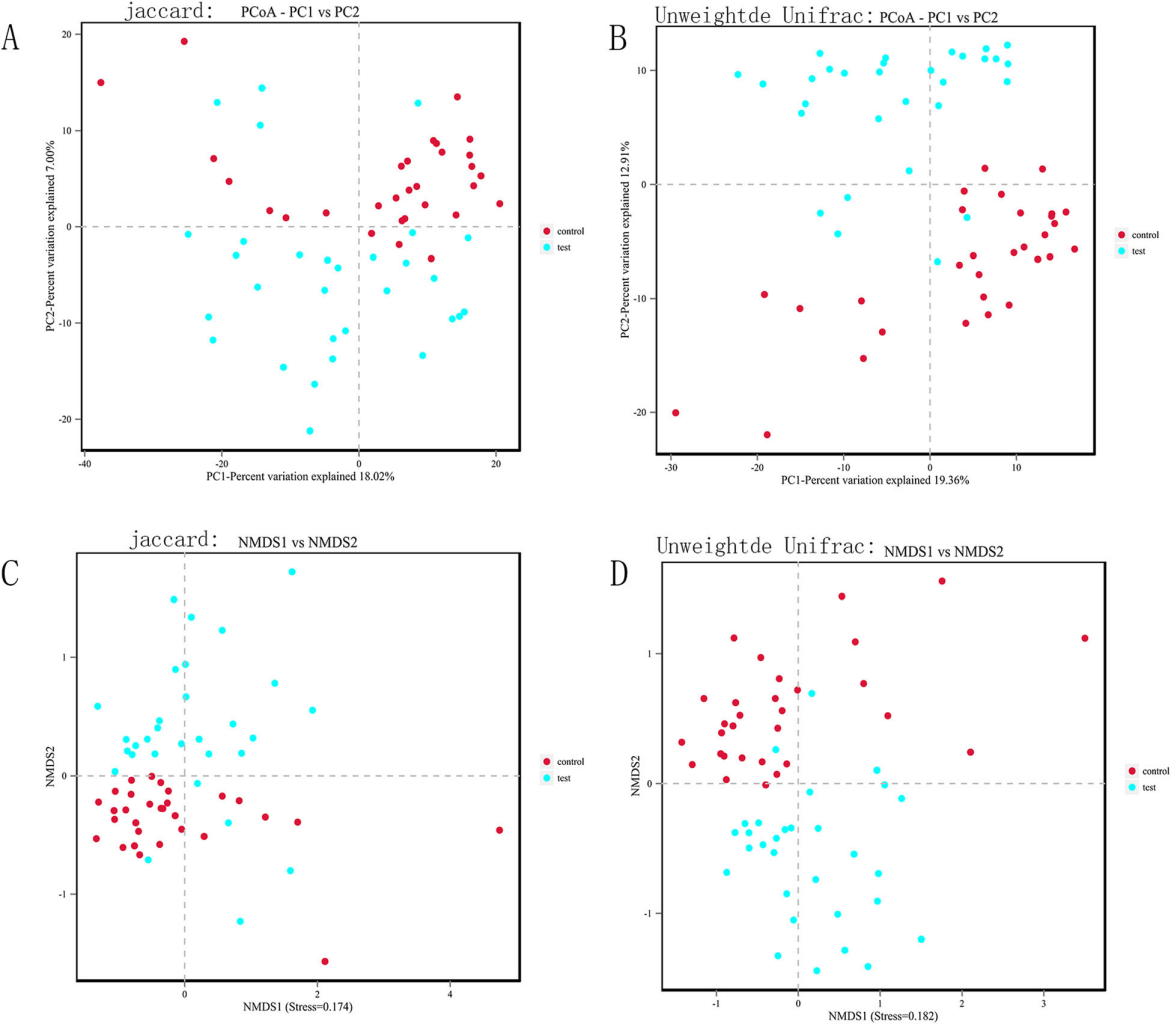
**a** Principal coordinates analysis (PCoA, Jaccard) of the microcosm composition. **b** Principal coordinates analysis (PCoA, unweighted UniFrac) of the microcosm composition. **c** Non-metric multi-dimensional scaling analysis (NMDS, Jaccard) of the microcosm composition. **d** Non-metric multi-dimensional scaling analysis (NMDS, unweighted UniFrac) of the microcosm composition

The β-diversity of the gut microbiota in the two groups was measured by principal coordinates analysis (PCoA) and non-metric multi-dimensional scaling (NMDS) analysis, which show the degree of similarity between the two microbial communities. The results revealed significant differences in species presence and evolution between the two groups (Fig. 3).

### Abundance and structural changes in gut microbiota at different levels in the gallstone group

**Fig. 4:**
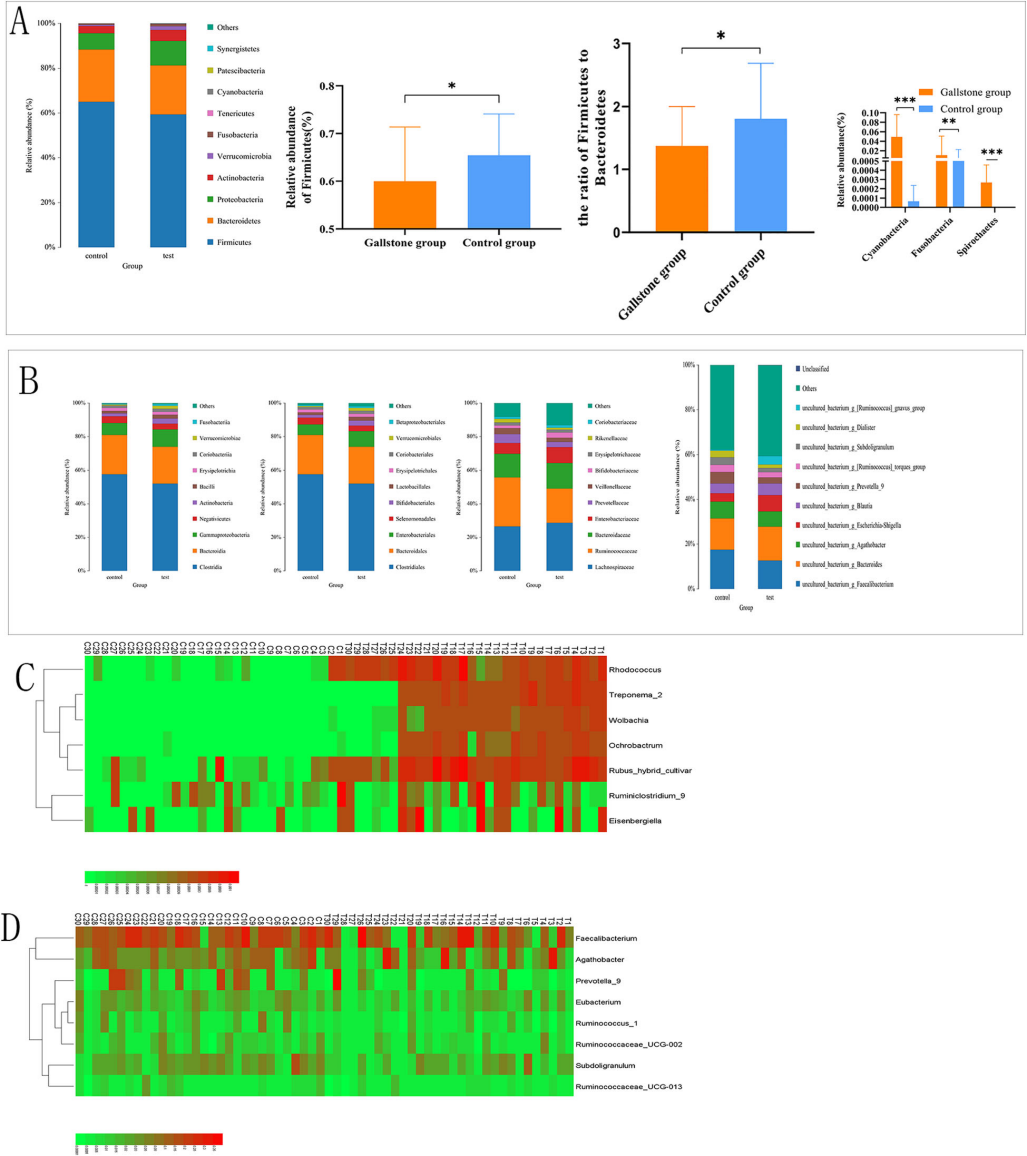
**a** At the phylum level, the number of *Firmicutes* was significantly reduced in the gallstone group, and the *Firmicutes*/*Bacteroidetes* (F/B) ratio was also significantly decreased. *Cyanobacteria*, *Fusobacteria*, and *Spirochaetes* were significantly increased in the gallstone group. **b** The structure and relative abundance of the flora in the two groups at the class, order, family, genus, and species levels. **c** At the genus level, the gallstone and control groups were compared, and the bacteria with significantly increased abundance are shown. **d** The bacteria with significantly reduced abundance are shown. Data are expressed as the mean ± SD, *p < 0.05, **p < 0.01, ***p < 0.001, ns > 0.05. Statistically significant differences (p < 0.05) between the two groups were determined by Mann-Whitney U test

At the phylum level, the major bacterial phyla in both groups were *Firmicutes*, *Bacteroidetes*, and *Proteobacteria*. These 3 major phyla accounted for 95.6% of the bacteria in the control group and 92.0% in the gallstone group, and the most abundant phylum in both groups was *Firmicutes*. However, compared with the control group, the number of *Firmicutes* was significantly reduced in the gallstone group. In the gallstone group, the *Firmicutes*/*Bacteroidetes* (F/B) ratio was also significantly decreased. *Cyanobacteria*, *Fusobacteria*, and *Spirochaetes* were significantly increased in the gallstone group (Fig. 4a). The structure and relative abundance of the flora in the two groups were shown at the class, order, family, genus, and species levels (Fig. 4b). At the genus level, the gallstone and control groups were compared, and the bacteria with significantly increased abundance (Fig. 4c) and the bacteria with significantly reduced abundance (Fig. 4d) were determined and are shown in the figures.

### LEfSe analysis between groups

**Fig. 5:**
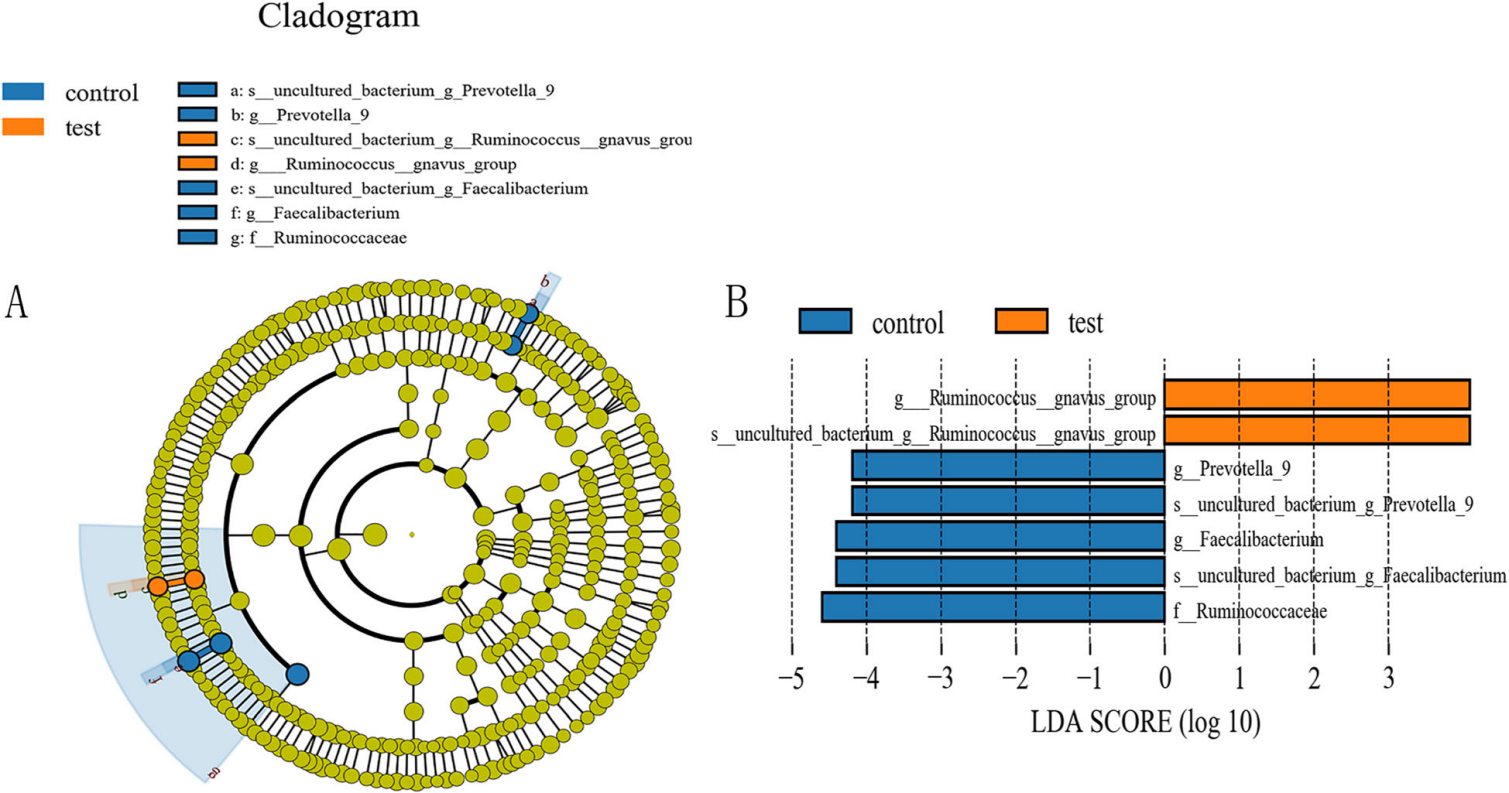
Different structures of gut microbiota in the test and control groups by LEfSE analysis. **a** LEfSe cladogram in orange shows the taxa enriched in the gallstone group and in blue shows the taxa enriched in the control group. The diameter of each circle is proportional to the abundance. **b** Specific phylotypes of gut bacteria in response groups using LEfSe. The histogram shows the LDA scores computed for features at the OTU level. The lateral text shows the taxonomic profiles of all the OTUs, which were significantly different between the test and control groups

LEfSe analysis aided in determining the biomarkers that were significantly differentially present among the different groups. The figure generated by the LEfSe analysis showed the taxonomic groups with the largest difference between the two groups at different levels (Fig. 5a). The histogram showed the observed differences in the 7 phylotypes between the gallstone and control groups (Fig. 5b). At the genus level, 3 genera could be used as biomarkers to distinguish between the control group and the gallstone group. In the gallstone group, *Ruminococcus gnavus* could be used as a biomarker, while in the control group, *Prevotella 9* and *Faecalibacterium* could be used as biomarkers.

### Abundance changes in gut microbiota with BSH activity at different levels in the gallstone group

**Fig. 6:**
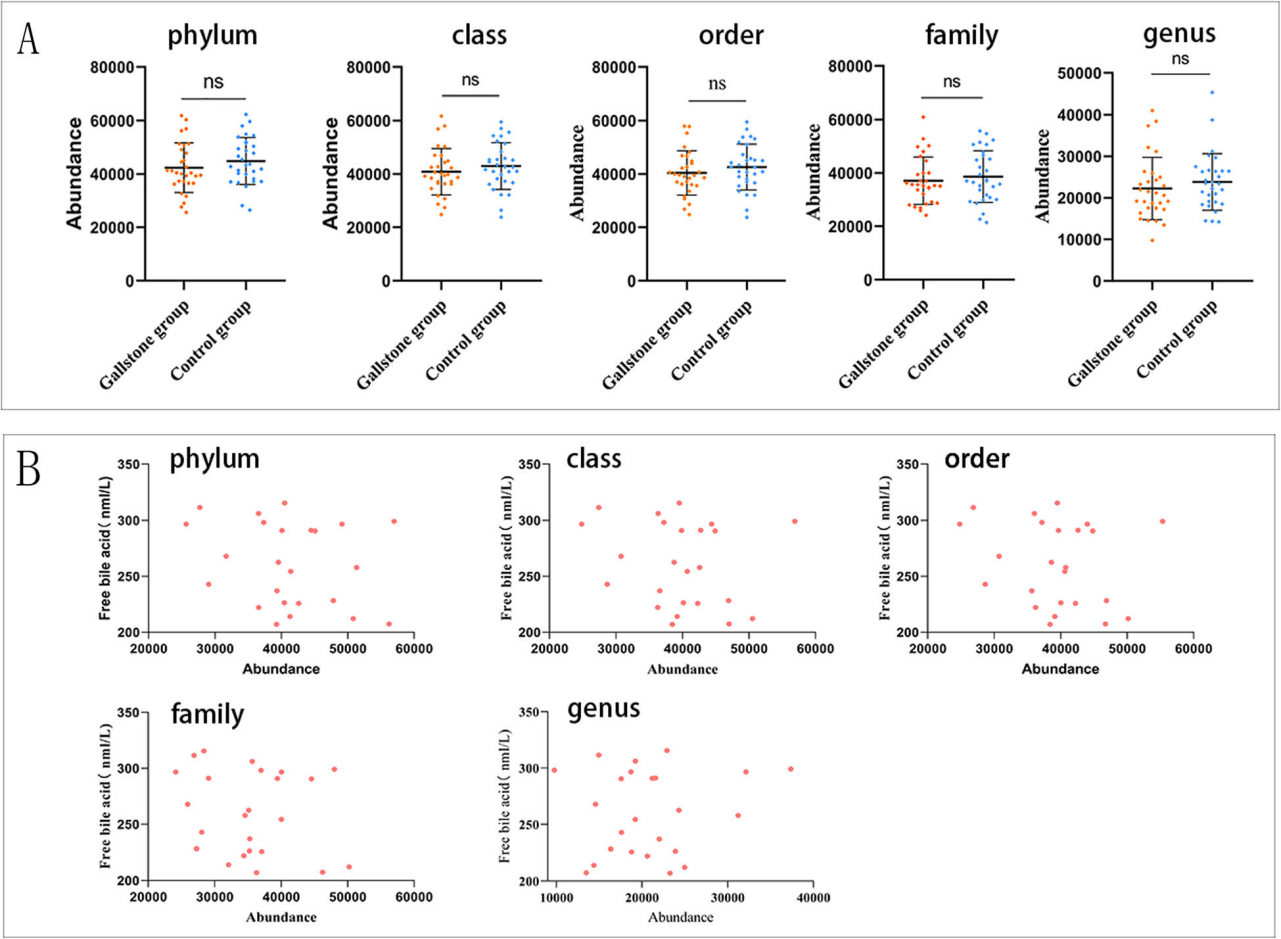
**a** At the phylum, class, order, family and genus levels, the abundance of bacteria with BSH activity was reduced in the gallstone group compared with the control group, but the difference was not statistically significant. **b** At different levels, the abundance of bacteria with BSH activity in the gallstone group was not correlated with free bile acids. Data are expressed as the mean ± SD, *p < 0.05, **p < 0.01, ***p < 0.001, ns > 0.05, Statistically significant differences (*p* value< 0.05) between the two groups were determined by Mann-Whitney U test

**Fig. 7:**
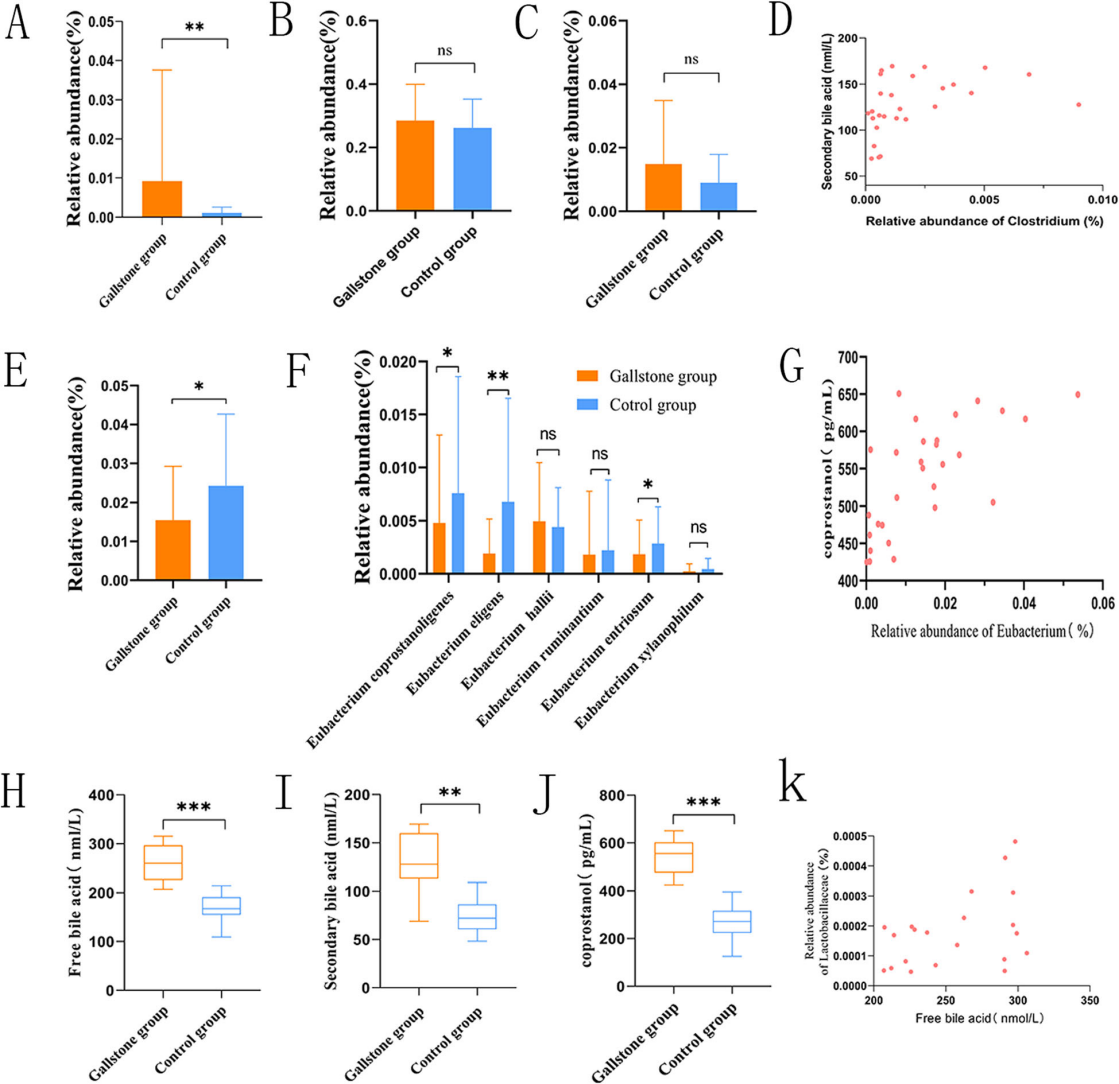
**a** The relative abundance of *Clostridium* was significantly increased. **b** The abundance of *Lachnospiraceae* was not significantly increased. **c** The abundance of *Peptostreptococcaceae* was not significantly increased. **d***Clostridium* was positively correlated with secondary bile acids (Spearman’s correlation, *p* < 0.01, r = 0.609, *n* = 29). **e** The abundance of *Eubacterium* was significantly decreased in the gallstone group. **f** At the species level, the relative abundance of species. **g***Eubacterium* was positively correlated with coprostanol (Spearman’s correlation, *p* < 0.01, r = 0.663, *n* = 29). **h** A significant increase in intestinal free bile acids was observed in the gallstone group. **i** The results showed a significant increase in secondary bile acids in the gallstone group. **j** This study showed that coprostanol was significantly higher in the gallstone group than in the control group. **k** The *Lactobacillus* abundance was positively correlated with the free bile acid concentration (Spearman’s correlation, *p* < 0.05, r = 0.457, *n* = 29). All data are expressed as the mean ± SD, *p < 0.05, **p < 0.01, ***p < 0.001, ns > 0.05, Statistically significant differences (*p* value < 0.05) between the two groups were determined by Mann-Whitney U test in A, B, C, E, F, and H. Statistically significant differences (p value < 0.05) between groups were determined by Student’s t test in Fig. 7 I and J

Using the literature and databases, the gut microbiota with bile salt hydrolase (BSH) activity were screened at different levels. The results showed that at the phylum, class, order, family and genus levels, the abundance of bacteria with BSH activity was reduced in the gallstone group compared with the control group, but this difference was not statistically significant (Fig. 6a). At these different levels, the abundance of bacteria with BSH activity in the gallstone group was also not correlated with free bile acids (Fig. 6b). Interestingly, a significant increase in intestinal free bile acids was observed in the gallstone group (Fig. 7h). Bile salt hydrolase activity was not related to the abundances of BSH-active bacteria.

### Relationship between abundance changes in 7α-dehydroxylating gut bacteria and secondary bile acids

Enzymes involved in 7α-dehydroxylation are encoded by the bile acid-inducible gene (*bai*). Currently only the *Clostridium* genus and some strains of *Lachnospiraceae* and *Peptostreptococcaceae* are known to have 7α-dehydroxylation activity, and these microbes account for only a small portion of the gut microbiota. This study demonstrated a significant increase in *Clostridium* in the gallstone group (Fig. 7a), while the abundance of *Lachnospiraceae* and *Peptostreptococcaceae* was not significantly increased (Fig. 7b, c). The results also showed a significant increase in secondary bile acids in the gallstone group (Fig. 7i), and *Clostridium* was positively correlated with secondary bile acids (Fig. 7d).

### Abundance changes in intestinal bacteria with cholesterol-lowering effects

Small amounts of cholesterol-lowering strains can be isolated from animal faeces (7, 12). All these strains exhibit similar properties and are currently attributed to the *Eubacterium* genus. In this study, compared with the control group, the abundance of *Eubacterium* was significantly decreased in the gallstone group (Fig. 7e). At the species level, the relative abundance of coprostanoligenic species (Fig. 7f) was significantly reduced. Moreover, coprostanol levels were significantly higher in the gallstone group than in the control group (Fig. 7j), and *Eubacterium* was positively correlated with coprostanol (Fig. 7g). Coprostanol is a conversion product of cholesterol. The abundance of these cholesterol-catalysed bacteria may result in reduced cholesterol conversion and increased cholesterol absorption.

## Discussion

Wang et al. [3] showed that gut microbiota at different levels changed with gallstone formation, indicating that gut microbiota dysbiosis plays an important role in the promotion of gallstone formation. Fremont-Rahl et al. found that compared to the control group, the incidence rate of cholesterol gallstones in sterile mice fed LD significantly increased. They believed that changes in gut microbiota could increase the occurrence of cholesterol gallstones by changes in gallbladder movement, local inflammation, and increases in mucin gene expression and mucin gel accumulation [22]. Our data also indicated that gallstone formation was associated with gut microbiota dysbiosis. In the gallstone group, the diversity of intestinal bacteria and the abundances of certain phylogroups significantly decreased (Fig. 4a), especially *Firmicutes*, the largest phylum in the gut microbiota, while the relative abundances of *Cyanobacteria*, *Fusobacteria*, and *Spirochaetes* significantly increased. In contrast to sterile mice, although the diversity and abundances of intestinal bacteria in patients with gallstones were significantly decreased, they were not completely free of bacteria. Therefore, we suggest that gut microbiota may affect the occurrence of cholesterol gallstones through other pathways. Carolien et al. disturbed the gut microbiota of mice by feeding them antibiotics, which significantly reduced the diversity and relative abundances of intestinal bacteria. As a result, the expression of Asbt, inhibited by Gata4, was changed, the absorption of bile acid in the intestinal tract was increased, and the synthesis of bile acid in the liver was reduced [23]. Bile acid is a potent activator of intestinal FXR, especially chenodeoxycholic acid (CDCA) [24]. After intestinal FXR is activated, intestinal epithelial cells release fibroblast growth factor 19 (FGF19), which inhibits the liver enzyme CYP7A1 (cholesterol 7α-hydroxylase) through the portal vein [25–27]. Duan et al. showed that the increase in bile acid uptake by intestinal epithelial cells could inhibit CYP7A1 through the FFX-FGF19 pathway. Since CYP7A1 is a key enzyme in cholesterol elimination, the inhibition of CYP7A1 could cause the accumulation of cholesterol in the liver [28]. Decreases in gut microbiota in patients with gallstones can increase the hepatic cholesterol secretion [24], which may lead to oversaturation of bile cholesterol and induction of cholesterol stone formation. Of course, this hypothesis has not been confirmed, and further validation is needed.

The intestinal bacteria uncouple the bound bile acid into free bile acid through BSH and then convert the free bile acids into secondary bile acids through 7α-dehydroxylation [29]. Currently, only the *Clostridium* genus [30] and some strains of *Lachnospiraceae* and *Peptostreptococcaceae* [31] in intestinal bacteria are known to have 7α-dehydroxylation activity, and these bacteria are known as 7α-dehydroxylating bacteria [32]. 7α-Dehydroxylating bacteria have bile acid-inducible (*bai*) genes, which encode 7α-dehydroxylase enzymes. There are 3 key enzymes in this pathway, which are encoded by *baiA2*, *baiB*, and *baiE* and produce the bile acid 3α-hydroxysteroid dehydrogenase, bile acid coenzyme A-linked enzyme, and bile acid 7-alpha dehydrase, respectively [33]. In the presence of cholic acids, the expression of *bai* can be upregulated [32].

The abundances and diversity of 7α-dehydroxylating bacteria in the intestinal tract of patients with gallstones are poorly understood, and these bacteria account for a small portion (< 1%) of the total gut microbiota. However, the effects of these low-abundance gut microbiota on the host may be enormous, and they can increase the secondary bile acids in the intestinal tract, such as deoxycholic acid (DCA) and lithocholic acid (LCA). These secondary bile acids return to the liver through the enterohepatic circulation. Because 7α-hydroxylation does not occur with secondary bile acids in the human liver, bile acids can accumulate to a very high level in human bile [34]. In contrast, 7α-hydroxylation can occur on secondary bile acids in rodents to maintain the hydrophilic bile acid pool size [35].

The formation of secondary bile acids by gut microbes has been a topic of great biomedical significance. 7α-Dehydroxylating bacteria convert primary biliary acids to secondary bile acids to inhibit the growth of *Clostridium difficile* in the body. Monocytes/macrophages, dendritic cells, and natural killer T cells express the functional bile acid receptor FXR. Under physiological concentrations, bile acids, especially hydrophilic secondary bile acids, play a role in the assembly of NLRP3 inflammasomes as negative regulators, and this effect requires functional FXR [36, 37]. However, in the presence of CDCA, DCA and LCA can inhibit FXR [38]. Our study found an increase in free bile acids and secondary bile acids in the enterohepatic circulation, which may lead to the inhibition of functional FXR in immune cells. This will promote the release of inflammatory cytokines, which will cause local inflammation.

GPBAR1 is a bile acid receptor that is expressed by innate immune cells, macrophages, and natural killer T cells and is considered necessary to maintain immune homeostasis in the intestine and liver [39–41]. In mice and humans, GPBAR1 is also abundant in epithelial cells of the gallbladder and gallbladder smooth muscle. The activation of GPBAR1 in epithelial cells promotes bile secretion, but the activation of the GPBAR1 receptor in smooth muscle cells leads to relaxation of smooth muscle, resulting in gallbladder filling [42]. Application of an endogenous or synthetic TGR5 ligand significantly increases gallbladder volume by more than two-fold [43]. The secondary bile acids DCA and LCA are potent GPBAR1 agonists.

The increase in the concentration of secondary bile acids promotes gallstone formation. Our data showed that the abundances of 7α-dehydroxylating bacteria, such as *Clostridium*, *Lachnospiraceae* and *Peptostreptococcaceae* all increased, especially *Clostridium*. We detected significant increases in secondary bile acids in patients with gallstones. The secondary bile acid content was positively correlated with the relative abundance of *Clostridium*. Berr et al. showed that the increase in secondary bile acids promoted the formation of cholesterol gallstones [44–46]. Our finding of a significantly increased abundance of *Clostridium* is consistent with the study by FRIEDER et al. [44]. However, the increases in *Lachnospiraceae* and *Peptostreptococcaceae* were not significant, which may be related to the increase in free bile acids in the intestine.

BSH-active bacteria can convert bound bile acids into free bile acids. Our study showed that the intestinal free bile acids significantly increased in the gallstone group (Fig. 7h), indicating that intestinal BSH activity was significantly increased. We analysed the abundances of BSH-active bacteria and found that the abundances of BSH-active bacteria at different levels were not significantly different between the control group and the gallstone group, indicating that BSH activity was not related to the abundances of BSH-active bacteria [47]. Ziwei et al. detected the enzyme activity of different BSH systems and found that BSH-T3 showed the highest enzyme activity, which was only found in *Lactobacillus* [21].

Our study also indicated that the *Lactobacillus* abundance was positively correlated with free bile acid concentration (Fig. 7k). Ridlon et al. and others showed that free bile acids can inhibit intestinal bacteria and regulate gut microbiota structure [31, 48, 49]. Our data showed that free bile acids were negatively correlated with chloroplasts. Previous reports have shown that many strains of the *Eubacterium* genus can convert intestinal cholesterol to coprostanol and that coprostanol is excreted in the faeces, thereby reducing cholesterol absorption. Only a few intestinal bacteria with cholesterol-lowering effects have been identified. They have similar properties, and most of them belong to the *Eubacterium* genus. Interestingly, our study showed that the *Eubacterium* genus in the gallstone group was significantly lower than that in the control group. The conversion of cholesterol into coprostanol in the human intestinal tract is mainly performed by a large number of coprostanoligenic strains belonging to the *Eubacterium* genus. Our data also showed that coprostanoligenic strains were significantly decreased in the gallstone group. This indicates that gallstone formation may be related to the reduction in *Eubacterium* because the reduction in intestinal bacteria with cholesterol-lowering effects causes the increased absorption of cholesterol and the increased secretion of hepatic cholesterol. Notably, *Fusobacteria* significantly increased in the gallstone group and was negatively correlated with coprostanoligenic strains, indicating that *Fusobacteria* may have an inhibitory effect on the growth of coprostanoligenic bacteria. LEfSe analysis indicated that the *Ruminococcus gnavus* group could be used as a biomarker to distinguish the gallstone group from the control group. From the proximal end to the distal end, the intestinal environment changes with different segments of the intestine. One potential limitation of this study is that the fecal microbial composition may not reflect small intestinal microbiota.

## Conclusion

In summary, we studied the structure and function of the gut microbiota in patients with gallstones and the relationships between changes in abundance in intestinal bacteria and bile acids and cholesterol in patients with gallstones. We conclude that intestinal flora imbalance affects bile acid and cholesterol metabolism and is associated with gallstone formation. Our findings warrant further exploration of the correlation between gut microbiota dysbiosis and gallstone formation, which holds great significance in understanding the mechanism of gallstone formation.

Intestinal microbiological disorders are closely related to the formation of gallbladder stones.

## Supplementary information

**Additional file 1.** Sequencing data quality assessment.

## Data Availability

The relevant raw data from this study can be readily available upon request for non-commercial purposes per a request from the corresponding author.

## Abbreviations

16S rRNA: *16S* ribosomal RNA
Asbt: Apical sodium-dependent bile salt transporter
bai: Bile acid-inducible
BMI: Body Mass Index
BSH: Bile salt hydrolase
CDCA: Chenodeoxycholic acid
CYP7A1: Cholesterol 7α-hydroxylase
DCA: Deoxycholic acid
ELISA: Enzyme-linked immunosorbent assay
F/B: Firmicutes/Bacteroidetes
FGF19: Fibroblast growth factor 19
FXR: Farnesoid X receptor
GPBAR1 or TGR5: G-protein-coupled bile acid receptor 1
LCA: Lithocholic acid
LD: Lithogenic diet
LDA: Line discriminant analysis
MRCP: Magnetic resonance cholangiopancreatography
NMDS: Non-metric multi-dimensional scaling
OD: Optical density
OTUO: Operational taxonomic unit
PCoA: Principal coordinate analysis
PCR: Polymerase chain reaction
PXR: Pregnane X receptor
RSA: Rank species abundance
SD: Standard deviation
VDR: Vitamin D receptor
